# Digital Therapeutics for the Egocentric and Allocentric Neglects in Patients with Brain Injury: A Mini Review

**DOI:** 10.3390/brainsci13081170

**Published:** 2023-08-06

**Authors:** Woo-Hyuk Jang, Sang-Min Seo

**Affiliations:** 1 Department of Occupational Therapy, Kangwon National University, Samcheok 25949, Republic of Korea; wlqtksek@hanmail.net; 2 Department of Occupational Therapy, Semyung University, Jecheon 27136, Republic of Korea

**Keywords:** egocentric neglect, allocentric neglect, digital therapeutics, apple cancellation test, broken hearts test

## Abstract

Various therapeutic approaches have been developed for neglect. Many studies have demonstrated the effect of digital therapeutics (DTx) on neglect. However, few studies have reported the effects of DTx on egocentric and allocentric neglect. The differentiation of types of neglect and separate interventions is crucial in the rehabilitation process. In this article, seven studies on DTx on egocentric and allocentric neglect were reviewed. DTx, which was employed in these studies, could be classified as follows: (1) software adaptation in traditional treatment, (2) VR game using the head-mount display as treatment, and (3) the development of a new digital program like ReMoVES. In addition, more studies and more effective results were reported for egocentric neglect than for allocentric neglect. In future studies, each effect on egocentric and allocentric neglect should be identified in detail with the appropriate use of differential evaluation and long-term application of independent DTx.

## 1. Introduction

Neglect is a clinical feature observed in cases of brain injury, particularly those affecting the right hemisphere, in which individuals have difficulty recognizing the contralesional side of space [1,2]. According to previous studies, patients with neglect tend to have poorer rehabilitation outcomes [3], longer hospital stays [4], and are less likely to be discharged to their homes [5] than patients without neglect. Consequently, neglect has been identified as a significant predictor of decreased levels of functional independence [6], increased utilization of healthcare resources [7], and increased burden on the patient’s family [8]. These findings highlight the detrimental effects of neglect on various aspects of patient outcomes.

Neglect has been divided into various subtypes according to classification criteria because of its heterogeneous characteristics [9,10]. For example, neglect is classified as sensory, motor, and representational (imaginal) neglect. In addition, in terms of the spatial domain, neglect is classified as personal neglect, peri-personal neglect, and extra-personal neglect. In terms of the frame of reference, neglect is categorized as egocentric neglect and allocentric neglect (Table 1) [9,10].

The frame of reference is defined as the process in which the brain accepts information and identifies the up–down, left–right, front–back directions, and the relative scale of each axis by specifying the central location [11]. The brain receives information differently depending on whether the central criterion is a viewer or an object. Therefore, the symptoms of neglect are also very different [12]. Egocentric neglect is characterized by responses missing on the contralateral side with respect to the viewer. In contrast, allocentric neglect is characterized by responses missing on the contralateral side with respect to the object [13]. Because of these different characteristics of the two types, allocentric neglect may have a more negative impact on participation in activities of daily living than egocentric neglect [14]. A differential diagnosis between allocentric neglect and egocentric neglect has become possible with the development of evaluation tools, such as the Apple Cancellation Test and the Broken Heart Test [15,16]. In general, these evaluations are performed by counting the number of correct (target of complete shape) and incorrect (one of the left and right targets is incomplete) responses after the patient has marked the target of a complete shape [15,16]. Egocentric neglect is determined by the asymmetry score (commission error) between the correct answers on the right and the left based on the center of the evaluation paper. On the other hand, allocentric neglect is discriminated by the asymmetry score (omission error) between the incorrect answer on the right and the wrong answer on the left based on the target in all incorrect answers [15,16]. The criteria for diagnosing egocentric (cut-off ≥ 3) and allocentric neglect (cut-off ≥ 2) are the same in the Apple Cancellation Test and the Broken Heart Test [15,16]. Egocentric and allocentric neglect, which have been reported on relatively recently, have been mentioned by many scholars as requiring differential diagnosis and intervention according to the type [17].

As described above, because neglect is closely related to functional outcomes, various therapeutic approaches have been developed for both egocentric and allocentric neglect. These include visual scanning training, eye patching, caloric vestibular stimulation, visuomotor imagery, mirror therapy, transcutaneous electrical nerve stimulation (TENS), optokinetic stimulation, constraint-induced movement therapy, prism adaptation, transcranial direct current stimulation (tDCS) activity of daily living (ADL) training, repetitive transcranial magnetic stimulation (rTMS), and digital therapeutics [18,19,20]. A previous study reported that the existing therapeutic modalities were ineffective in treating allocentric neglect [21]. Therefore, some therapeutic approaches for allocentric neglect have been attempted as follows: repetitive transcranial magnetic stimulation (rTMS) [22], visual cueing using immersive virtual reality [23], and computer-based training during transcranial direct current stimulation (tDCS) [24].

Digital therapeutics (DTx) is a newly emerging concept of therapeutic approach in the healthcare system [25]. It is a subdivision of digital health, which is defined as a healthcare system driven by any form of digital technology [26]. The components of digital therapeutics include smartphones, personal digital assistants, virtual reality (VR), and tablet computers that converge with software algorithms [19]. DTx can help reduce healthcare costs [27] and improve availability to patients [28]. In addition, DTx can combine treatment and prevention simultaneously because this approach can provide 24 h monitoring which was previously impossible [29]. As a result, many DTx have been introduced in the neuro-rehabilitation field, and the clinical effects have been demonstrated [20]. Many studies have demonstrated the effect of DTx on neglect [10,18,19,20]. In addition, many reports have been published on the therapeutic potential of neglect using VR, HMD, etc. [30,31]. However, most of the studies only investigated the effects of the intervention without differentiating the types of neglect. In contrast, there are few studies on DTX after differential diagnosis that are essential for a selective approach according to the type of neglect [23,24,32,33,34]. This review examined DTx for egocentric and allocentric neglect in patients with brain injury. Therefore, this review included a differential diagnosis between egocentric and allocentric neglect and studies that investigated the effects of DTx according to the type of neglect involved.

## 2. Materials and Methods

**Figure 1:**
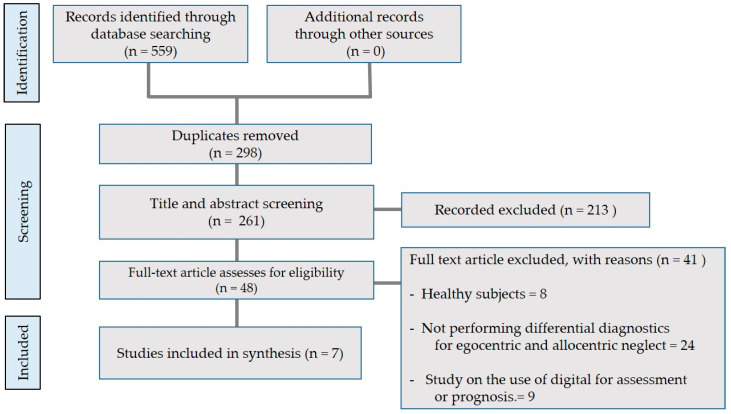
Flow diagram for the selection of studies for review.

This paper reviews DTx studies on frames of reference in neglect (egocentric neglect and allocentric neglect) related to brain injuries. Relevant studies from 1980 to 2023 were identified by accessing the following electronic databases: Web of Science, PubMed, and Google Scholar. The following keywords were used to search the databases: neglect, apple test, heart test, egocentric neglect, allocentric neglect, digital therapeutics, and brain injury. This review was limited to studies involving human subjects with neglect. The relevant studies were selected based on the flow chart shown in Figure 1. A total of seven studies were selected and reviewed. Five studies were in medical journals, four of which were research studies [32,33,34,35], and one was a case study [24]. Two studies were in non-medical journals, both of which were conference papers [23,36] (Table 2).

## 3. Studies of Digital Therapeutics (DTx) for Egocentric and Allocentric Neglect

Turgut et al. [32] examined the effects of digital therapy (eye-tracking training based on a computer program) in 32 stroke patients with neglect. They classified the patients into two groups (experimental group, 16 patients, and control group, 16 subjects). The control group received only basic rehabilitation training, and the experimental group received rehabilitation training and eye tracking (digital therapeutics, DTx) at the same time. The eye tracking training consisted of two programs: (1) when a blue square on the computer screen moved in the direction of the neglect, and (2) when the square changed to red, training to press the switch was performed (20 min/one session, total of eight sessions over two weeks). The differential evaluation of egocentric and allocentric neglect was performed using the Apple Cancellation Test. This eye-tracking task was performed simultaneously with transcranial direct current stimulation (tDCS, 1.5 and 2.0 mA) of the right posterior parietal lobe (P4). The assessments were conducted as two pre (T1, T2)/two post (T3, T4) comparisons, and there were no significant changes in the Apple Cancellation Test and the three horizontal line bisection test in both groups at T1 and T2. However, the neuropsychological test (body orientation) and the clock drawing test conducted at the last assessment of the experimental group (T4, 6 days after the end of training) showed effective significant changes in both egocentric and allocentric neglect [32]. This study had several limitations. This study was not designed to observe the long-term effects and only the effects of DTx because digital therapy was applied with tDCS. In addition, there were two pre-training assessments for both groups, but there was no statistical analysis, and both groups underwent separate rehabilitation during the intervention period.

Hagiwara et al. [23] reported the effects of the training to provide visual cues using virtual reality (VR) for treating allocentric neglect in four stroke patients. The visual cues were provided using a head-mount display (HMD) type VR, Oculus Rift (Oculus VR., Inc. 19800 MacArthur Blvd Irvine, CA 92612 United States). The training consisted of a four-digit reading task followed by four actions: (1) displaying the clue stimulation, (2) obscuring the surrounding environment, (3) moving the clue stimulation, and (4) removing the obscuration. This action drew the patient’s attention to the neglected side, and then a four-digit number was read again. This training was repeated 10 times. Evaluation was performed using the apple cancellation test and line bisection with three horizontal lines, twice before and after the intervention. Patients showed fewer errors in the Apple Cancellation and Line Bisection Tests after training than those before training [23]. On the other hand, this study was conducted only once. Therefore, the author could only demonstrate the immediate effects of the treatment. Thus, the long-term effects of the treatment were not reported. The small number of subjects was another limitation. Furthermore, although we used the Apple Cancellation Test, which can be used for differential diagnosis, we cannot understand egocentric neglect by analyzing only the results for commission.

Huygelier et al. [33] investigated the effect of therapy using a VR game on stroke patients with neglect. They classified the subjects into two groups (experimental group, seven stroke patients with neglect; control group, 15 normal subjects). Both groups underwent training via VR using an Oculus Rift head-mounted display (HMD) VR device (Oculus VR., Inc.). The VR training included a variety of contents for treating neglect, including stimulation and cueing on the neglected side. Six sessions (45 min/1 session/day) were performed over seven days. The assessments were the heart cancellation test in the psychometric properties of the Dutch Oxford Cognitive Screen (OCS-NL) and the letter cancellation test and figure copy task in the Behavioral Inattention Test (BIT) were administered before and after the intervention. The authors reported that presenting various stimuli and cues in the neglected area using VR was effective in accurate target recognition in egocentric neglect patients [33]. On the other hand, their study was limited by the short period (seven days) and the use of distinguishable evaluation tools (to determine whether neglect is present). In addition, although this study used the heart cancellation test of OCS-NL, which can distinguish between types of neglect, we could only indirectly check the effect on egocentric neglect by counting only omissions (the number of commissions was used to determine allocentric neglect).

In 2020, Trombini et al. [36] reported the effects of computer-assisted cognitive training (CCT) using the Remote Monitoring Validation Engineering System (ReMoVES: University of Genova) (ReMoVES, Touch screen method) for the treatment of neglect in two patients (patient A; posterior cortical atrophy, patient B; right cerebral hemorrhage). Training with ReMoVES and tDCS (1.5 mA) on the right posterior parietal lobe (P4) was provided simultaneously. For CCT training, both patients were trained using the computerized Albert Test and Apple Cancellation Test in ReMoVES (20 min/session, five times/week for two weeks). The assessment used the computerized Albert Test, and the Apple Cancellation Test used for training was used, with the addition of a line bisection test (paper and pencil method). In patient A, only a significant improvement in egocentric neglect was confirmed using the Albert test, whereas, in patient B, a significant improvement in both egocentric and allocentric neglect was confirmed using the line bisection test, the Albert Test, and the Apple Cancellation Test [36]. There was no significant difference in the drawing test in both subjects. On the other hand, the authors did not exclude the learning effect by conducting training using evaluation tools (computerized apple cancellation test and Albert test). And a separate analysis of the effects of CCT by combining tDCS during a short training period cannot be performed. In addition, there was no control group and long-term effect data. Finally, the tool used in the evaluation, the line bisection test, is not described in detail, making it difficult to identify the exact tool.

Schenke et al. [35] reported on the effects of digital therapy (auditory cueing and computer-based eye-tracking training) in stroke patients with egocentric neglect. This study consisted of Study 1 and Study 2. In Study 1, they classified the patients with neglect into two groups (experimental group, 11 patients; control group, 14 patients). The experimental group underwent auditory cueing with basic rehabilitation. The control received only basic rehabilitation. This study involved training detect to the movement of sound from the right to left headphones while wearing headphones (30 min/session, five times/week for three weeks). In Study 2, another eight patients with neglect (one group) were subjected to computer-based training (digital therapeutics), which provided patients with auditory cueing and eye-tracking training simultaneously. In this study, the patients had to press a button in the following situations: (1) when the target moved from right to left on the computer screen and (2) when they heard the word “here” through the headphones (30 min/session, five times/week for three weeks). In Study 1, the line bisection test (3 lines) was administered (twice) before and (twice) after training, and the Apple Cancellation Test was administered (twice) before and (once) after training. In Study 2, the visual scanning test was administered twice before and after training. After three weeks of treatment, both studies reported that the severity of egocentric neglect decreased on the line bisection, the Apple Cancellation Test, and the visual scanning test. In addition, Study 2 confirmed that digital therapeutics allowed for the simultaneous provision of auditory cues and eye-tracking training and that convenient treatment was possible [35]. On the other hand, they did not report the long-term effects of this DTx and control group in Study 2. In addition, there was no mention of allocentric neglect despite the use of the discriminative apple cancellation test.

In 2021, Vestito et al. [24] examined the effects of computer-assisted cognitive training (CCT) on egocentric neglect in one patient with posterior cortical atrophy. The four-week training consisted of CCT only for the first two weeks (T1) using the Remote Monitoring Validation Engineering System (ReMoVES: University of Genova). In this study, three of the most appropriate games for the treatment of neglect in ReMoVES, including ShelfCans, OwlNest, and ChinaLanterns, which did not provide detailed information about the games, were applied (20 min/session, five times/week for two weeks). During the next two weeks (T2), CCT and tDCS (P4, 1.5 mA on the right posterior parietal lobe (P4) were delivered simultaneously (20 min/session, five times/week, for two weeks). Evaluations were carried out using Albert’s Test, line bisection test (22 lines), and Apple Cancellation Test installed in ReMoVES. Three tests were performed: one before training (T0, once) and one each at the end of T1 and T2 training. We only report significant results for all tests performed after T2. Therefore, they reported that the simultaneous application of both modalities (CCT and tDCS) was more effective than only CCT alone [24]. This study used the Apple Cancellation Test for a differential diagnosis. On the other hand, there was no direct relationship between the changes in egocentric and allocentric neglect. Another limitation of this study was that the improvements in egocentric neglect could be identified indirectly by the difference in omission on the Apple Cancellation Test.

Turgut et al. [34] compared the effect of two DTx in sixty patients with neglect. In Study 1, 30 stroke patients (right hemisphere injury) were divided into an experimental group (20 patients with neglect) and a control group (10 patients without neglect). In Study 2, another 30 stroke patients (right hemisphere injury) were divided into an experimental group (17 patients with neglect) and a control group (13 patients without neglect). In both studies, computerized “three blocks of Posner’s covert shift of attention task (Posner task)” were used for DTx. The Posner task was performed by pressing a square on the 24-inch monitor, where the stimulus image was presented between the two blank squares on the monitor. The cue elicits a rapid response by predicting where the stimulus picture will appear. The task was performed in two sessions consisting of three blocks of 100 trials each, with two days between the sessions. Response time to a stimulus was measured. In Study 1, endogenous cueing (centrally presented arrows as cues) was provided. In Study 2, exogenous cueing (peripherally presented crosses were used as cues) was provided and compared. In both studies, response time was measured twice, three days apart. The tools used for evaluation are the Apple Cancellation Test and the line bisection test. They reported that response time and omissions decreased with endogenous cueing but not with exogenous cueing. Therefore, endogenous cueing was more effective for egocentric neglect [34]. This study had a short study period of 4 days to explore the feasibility of training with the Posner task. This suggests that a longer-term training study is needed. On the other hand, this study presented the average unilateral neglect type between groups using the Apple Cancellation Test, but the results only compared omission error and response time to identify egocentric neglect. Therefore, their study was limited by the lack of a detailed comparison of the rehabilitation effects according to neglect type. Finally, there is no detailed description of the evaluation tool used for line bisection, making it difficult to understand the exact tool.

## 4. Discussion

The identification of neglect types and separate interventions are very important. In addition, treatment with DTx will be of interest to future societies. This study conducted a review of seven studies on DTx for egocentric and allocentric neglect.

The DTx used in these studies could be classified as follows: (1) software adaptation in traditional treatment [32,34,35], (2) VR game using the head-mounted display as treatment [23,33], (3) the development of a new digital program such as ReMoVES [24,36], and (4) the simultaneous stimulation of tDCS during DTx [24,32,36]. And the tDCS used was 1.5 mA (1.5 to 2.0 mA only for reference No. 29), all anodes were located at P4 (posterior parietal lobe) of the injured hemisphere, and DTx was performed simultaneously for 20 min. The most common training duration for DTx was 20–30 min per session, 4–5 times per week, for 2–3 weeks [24,32,34,36]. Next, there was one study that trained for 45 min per session, six times per week, for one week [33], and other studies conducted a before-and-after comparison study with one training session [23,34]. This suggests that long-term studies with DTx and follow-up tests are needed to determine whether the effects are sustained. In addition, while it is good to utilize existing training, we believe that research is needed to incorporate new equipment or expand into new approaches such as telerehabilitation. In these cases, it is believed that the cost and availability of equipment, lack of instruction clarity, and technological awareness should be considered, as suggested by studies on telerehabilitation utilizing virtual reality (VR) for neglect treatment [37,38]. 

Regarding the effectiveness of DTx, four studies showed effectiveness on egocentric neglect [24,33,34,35], two studies showed effectiveness on both egocentric and allocentric neglect [32,36], and only one study showed effectiveness on allocentric neglect [23]. On the other hand, when it comes to assessment tools for the differential diagnosis of neglect and the comparison of effects, the Apple Cancellation Test, a subtest of the Birmingham Cognitive Screen (BCoS), was most commonly used [23,24,32,34,35], and the Heart Cancellation Test, a subtest of the “psychometric properties of the Dutch Oxford Cognitive Screen (OCS-NL)”, was used in one study [33]. Some studies have used the Apple Cancellation Test in a computerized manner rather than the traditional paper-and-pencil method [24,36]. However, despite being a differential diagnostic assessment, two studies reported effects on both egocentric and allocentric neglect [32,36], while the others only found improvements in egocentric neglect (producing only omissions) [24,33,34,35] or allocentric neglect (producing only commissions) [23]. As such, research on allocentric neglect is still lacking, and although assessment tools with differential diagnosis were used, the fact that changes in egocentric neglect were identified by analyzing limited data is something that future studies should improve. It also shows that there is an urgent need to develop new intervention methods to improve allocentric neglect.

The next most commonly used assessment tool is the line bisection test. Most studies used a three-line (horizontal) assessment, which is a subtest of the Behavioral Inattention Test (BIT) [23,32,34], with only one study using a 22-line assessment [24], and the other study was unclear due to a lack of details [36]. Albert’s test was used in two studies [24,36], and the letter cancellation test and figure copy, which are subtests of the BIT, were used in one study [33]. Finally, the Clock Drawing Test was used in one study. Most of the tests were used to examine changes in egocentric neglect, and the clock drawing test was the only test used to examine changes in allocentric neglect besides the Apple Cancellation Test [32]. This suggests that there is a need to develop more diverse assessment tools for allocentric neglect and to differentiate between the two types.

Finally, we confirmed the effectiveness of DTx for neglect. In addition, a recent review article reported the effects of digital tools and approaches on motor functions (e.g., upper/lower extremity, balance, and gait) and language functions (e.g., aphasia) [37]. In addition, improvements in various cognitive functions, including memory, not just neglect, have been reported [37]. As such, the utilization of DTx has the advantages of reduced economic costs [27], increased patient availability [28], and continuous monitoring [29] compared to conventional methods. In addition, since training can be performed on devices such as smartphones and tablets, spatial constraints are reduced [19], and digital game-style training in telerehabilitation has been attracting attention for patient motivation because it is highly effective in stimulating interest [37,38]. With these many benefits and a wide range of applications, DTx is expected to become a rehabilitation tool for medical professionals and a new subject of research, including animal studies.

Nevertheless, there were some limitations in these studies. First, none of the studies reported the long-term effects of DTx [23,32,34,35,36]. Second, despite using an evaluation tool, such as the Apple Cancellation Test, which can distinguish between egocentric and allocentric neglect, it was used only to identify the presence or absence of neglect [24,33]. Third, some of the studies applied DTx with tDCS [24,32,36]. As a result, these studies could not demonstrate the pure effects of DTx. Fourth, the majority of the above studies did not demonstrate an effect on allocentric neglect. Nevertheless, it is important to note that these are studies that can be used for egocentric and allocentric neglect with DTx, which will be prominent in the future society. In addition, we believe that this review will serve as a basis for new DTx studies.

## 5. Conclusions

In this study, seven studies on digital therapeutics (DTx) for neglect (egocentric and allocentric) were reviewed. Therefore, in future studies, each effect on egocentric and allocentric neglect should be identified in detail with the appropriate use of differential evaluation and long-term application of independent digital therapeutics. Overall, more diverse studies on allocentric neglect will be needed. Finally, we look forward to further research with new developments and the effective utilization of DTx in a variety of ways for many healthcare professionals.

## Figures and Tables

**Table 1:** Subtypes of neglect.

Criteria	Aspect of Neglect	Range of Space	Frame of Reference
Subtype	Sensory neglect	Personal neglect	Egocentric neglect
	Motor neglect	Peri-personal neglect	Allocentric neglect
	Representational neglect	Extra-personal neglect	

**Table 2:** Digital therapeutics for egocentric and allocentric neglect.

Study	Sample Size (n)	Type of Brain Injury	Study Description	Frequency of Intervention	Follow Up	Outcome Measure for Effect	Results
Turgut et al. (2016) [32]	IC:16, CG:16	Cerebrovascular disease	Both groups received standard treatment IC: tDCS+ eye tracking task (DTx)	20 min/session (during tDCS)	8 sessions during 2 weeks	LBT, CDT, ACT, FIM.	IC group improved than CG in both egocentric and allocentric neglect
Hagiwara et al. (2018) [23]	IG: 4, CG: None	Stroke	IG: Visual cue using Oculus Rift (DTx) CG: N/A	Clinical trial in 1 session	None	ACT, LBT.	VR intervention has immediate improvement in allocentric neglect
Huygelier et al. (2020) [33]	IG:7, CG: 15	IG: Stroke CG: Normal subject	Both groups received the same VR gaming tasks using Oculus Rift (DTx)	45 min/session	6 sessions during 7 day	OCS-NL, BIT.	The feasibility of evaluation and therapy for egocentric neglect was confirmed
Trombini et al. (2020) [36]	2 Single- cases	Patient 1: PCA Patient 2: Right cerebral hemorrhage	A-tDCS + ReMoVES (DTx)	20 min/session, 5 days/week (during tDCS)	2 weeks	Albert’s test, ACT, in ReMoVES LBT.	Patient 1 Improvement of egocentric neglect was confirmed using Albert’s test score only Patient 2 Improvement of both types of neglect (egocentric and allocentric) was confirmed using the ACT as well as Albert’s test and LBT
Schenke et al. (2021) [35]	Study 1: IG:11, CG:14 Study 2: IG:18, CG:0	Stroke	Study 1 Both groups received standard treatment, IC: auditory cue Study 2 IG: computer-based training (=auditory cues + eye tracking) (DTx)	Study 1, 2 30 min/session, 5 sessions/week	Study 1, 2 3 weeks	Study 1: LBT, ACT. Study 2: visual scanning test.	Study 1, 2 IC group improved only in egocentric neglect
Vestito et al. (2021) [24]	1 Single-case	PCA	A-tDCS & A-tDCS + ReMoVES (DTx)	A-tDCS only 20min/session, 5 days/week A-tDCS + ReMoVES 20min/session, 5 days/week (during tDCS)	2 weeks 2 weeks	Albert’s test, LBT, ACT, in ReMoVES.	Mixed training (A-tDCS + ReMoVES) was effective for egocentric neglect
Turgut et al. (2021) [34]	Study 1: IG: 20, CG: 10 Study 2: IG: 17, CG: 13	Stroke IG: with neglect CG: without neglect	All groups received Posner Task (DTx) Study 1: Endogenous cues Study 2: Exogenous cues	Study 1,2 2 sessions	Study 1,2 two days between sessions	Study 1, 2: ACT.	Endogenous visuospatial tasks improve only in egocentric neglect

IC; intervention group, CG; control group, tDCS; transcranial direct current stimulation, DTx*;* digital therapeutics, LBT; Line bisection, CDT; clock drawing test, ACT; apple cancellation test, FIM; functional independent measure, BIT; behavioral inattention test, VR; virtual reality, OCS-NL; the Dutch Oxford Cognitive Screen, PCA; Posterior cortical atrophy, A-tDCS; anodal transcranial direct current stimulation, ReMoVES; the Remote Monitoring Validation Engineering System.

## Data Availability

The data used for this study are private but can be made available upon reasonable request.
